# Automatic segmentation of vestibular schwannomas from T1-weighted MRI with a deep neural network

**DOI:** 10.1186/s13014-023-02263-y

**Published:** 2023-05-08

**Authors:** Hesheng Wang, Tanxia Qu, Kenneth Bernstein, David Barbee, Douglas Kondziolka

**Affiliations:** 1 Department of Radiation Oncology, NYU Grossman School of Medicine, New York, NY 10016 USA; 2 Department of Neurosurgery, NYU Grossman School of Medicine, New York, NY 10016 USA

**Keywords:** Image segmentation, Vestibular schwannomas, Radiosurgery, Deep neural network, MRI

## Abstract

**Background:**

Long-term follow-up using volumetric measurement could significantly assist in the management of vestibular schwannomas (VS). Manual segmentation of VS from MRI for treatment planning and follow-up assessment is labor-intensive and time-consuming. This study aims to develop a deep learning technique to fully automatically segment VS from MRI.

**Methods:**

This study retrospectively analyzed MRI data of 737 patients who received gamma knife radiosurgery for VS. Treatment planning T1-weighted isotropic MR and manually contoured gross tumor volumes (GTV) were used for model development. A 3D convolutional neural network (CNN) was built on ResNet blocks. Spatial attenuation and deep supervision modules were integrated in each decoder level to enhance the training for the small tumor volume on brain MRI. The model was trained and tested on 587 and 150 patient data, respectively, from this institution (n = 495) and a publicly available dataset (n = 242). The model performance were assessed by the Dice similarity coefficient (DSC), 95% Hausdorff distance (HD95), average symmetric surface (ASSD) and relative absolute volume difference (RAVD) of the model segmentation results against the GTVs.

**Results:**

Measured on combined testing data from two institutions, the proposed method achieved mean DSC of 0.91 ± 0.08, ASSD of 0.3 ± 0.4 mm, HD95 of 1.3 ± 1.6 mm, and RAVD of 0.09 ± 0.15. The DSCs were 0.91 ± 0.09 and 0.92 ± 0.06 on 100 testing patients of this institution and 50 of the public data, respectively.

**Conclusions:**

A CNN model was developed for fully automated segmentation of VS on T1-Weighted isotropic MRI. The model achieved good performance compared with physician clinical delineations on a sizeable dataset from two institutions. The proposed method potentially facilitates clinical workflow of radiosurgery for VS patient management.

## Background

Vestibular schwannomas (VS), also known as acoustic neuroma, is a benign tumor which originates from the vestibular branch of the vestibulocochlear nerve in the internal auditory canal. VS usually grows slowly. However, a tumor growth can cause hearing loss, tinnitus, imbalance, and facial weakness. VS is the third most common nonmalignant primary brain tumor, accounting for about 6% of all intracranial tumors [1]. The incidence of VS in the USA is approximately 12 cases per million inhabitants every year [2].

The management options for VS include observation with regular imaging surveillance, surgical resection and radiosurgery [3]. Stereotactic radiosurgery (SRS), such as Gamma Knife radiosurgery (GKRS), is widely accepted as a safe and effective treatment option for VS [4]. A key step in the SRS workflow is that physicians delineate VS on MR images for treatment planning, which can be time-consuming. After the treatment, long-term imaging follow-up and assessment of tumor sizes are mandatory for clinical decision-making [2, 5]. While measuring the maximal linear dimension of a VS is recommended and commonly used to quantify tumor size [6], direct three-dimensional (3D) volumetric measurement would be a more accurate metric that allows detection of actual tumor growth [7]. Manually identifying and segmenting VS on serial MR during follow-up of up to years is labor-intensive, prohibiting its routine application in clinical practice [8, 9]. In comparison, automated method could improve the efficiency of detection and segmentation of brain tumors by 30.8% time saving [10]. Furthermore, manual contouring also tends to be user-subjective and highly variable among operators. Automatic segmentation of VS on MR could significantly ease the burden of manual operation, and improve VS management by providing accurate and reproducible volume measurements.

Deep learning (DL), especially convolutional neural networks (CNN), has been state-of-the art for a wide range of medical image applications [11]. Without hand-crafting features, DL-based methods have achieved remarkable performance improvements in many image segmentation tasks [12, 13]. In segmentation of VS, Shapey et al. employed a CNN model on anisotropic T1-weighted (T1W) and T2-weighted (T2W) MR images, and achieved a performance equivalent to human experts [14]. Lee et al. proposed a dual-pathway CNN to segment VS on T1W and T2W MR with a more nonuniform resolution [15]. The study demonstrated the feasibility of DL segmentation of follow-up MR for longitudinal analysis of VS after GKRS. Both networks utilized dedicated 2D layers on the axial image slices to exploit the high in-plane resolutions.

The workflow of GKRS and imaging follow-up that our institution uses in the last two decades acquires near-isotropic MR to minimize clinical time of the whole procedure. This study therefore developed a 3D CNN model to utilize the inter-slice and cross-slice information simultaneously to automatically segment VS on the MRI. Meanwhile, we included the publicly available MR and VS annotation dataset released by Shapey et al. [16] in model training and evaluation. With the sizable data from both institutions, the objective of this study was to establish a robust method to automatically segment VS on isotropic MRI, which will facilitate GKRS treatment planning and long-term monitoring of the tumor response after radiosurgery.

## Methods and materials

### Patient and data

Under the approval of the Institutional review board (IRB), 495 patients who received GK radiosurgery for VS between year 2012 and 2021 were enrolled in this retrospective study. The patients (Female/Male: 256/239) had a median age of 60 years in a range of 13 to 91 years old. The VS sizes varied from 0.03 to 17.75 cm^3^ with a median of 0.75 cm^3^. 74 patients (14%) had surgical resection of the tumor prior to the radiosurgery. The details of the patient population and tumor sizes were summarized in Table 1.

**Table 1:** Characteristics of patient and VS tumor

	Institutional Data	Public Data	Total Data
Patient Number	495	242	737
Sex(Male : Female)	239:256	95:147	334:403
Age (Median (Range))	60 (13–91)	56 (24–84)	NA*
Tumor Volume (cm^3^)	0.75 (0.03–17.75)	1.41 (0.04–10.78)	0.95 (0.03–17.75)
Data Split (Train : Validate : Test)	344:51:100	168:24:50	512:75:150
Train Volume (cm^3^)	0.77 (0.04–17.75)	1.36 (0.04–9.80)	0.98 (0.04–17.75)
Validation Volume (cm^3^)	0.75 (0.05–8.72)	1.86 (0.16–6.15)	1.06 (0.05–8.72)
Test Volume (cm^3^)	0.69 (0.03–11.58)	1.66 (0.07–10.78)	0.87 (0.03–11.58)

All the patients underwent MR imaging for treatment planning immediately after a Leksell stereotactic frame was fixed to the head [17]. The imaging examinations were performed on a Simens 1.5T or 3T MR scanner with an institutional protocol. T1-weighted contrast-enhanced MR images were acquired using the magnetization-prepared rapid acquisition with gradient echo (MPRAGE) sequence with TR/TE/TI of 4.15/2130/1100 ms and 2.35/2100/900 ms, respectively, for 1.5T and 3.0T scanning. The axial images had a 3D matrix of 256 × 256 × 208 with in-plane resolution of 0.82 × 0.82 mm and slice thickness of 1.0 mm. In the examination, a T2-weighted MR volume was also obtained with a low isotropic resolution (1.5–2.0 mm) to aid in tumor detection and delineation.

The gross tumor volume (GTV) for radiosurgery was manually contoured on the high-resolution T1W MR and reviewed by a team consisting of neurosurgeons, radiation oncologists and physicists. A GK treatment plan was subsequently designed to treat the volume without additional margin. A tumor margin dose in a range of 12 to 13 Gy was typically prescribed to the 50% isodose volume. All the contouring and treatment planning were performed in the GK treatment planning system (Leksell GammaPlan). The target volumes were exported from the system as the ground truth of VS for the MR to develop the automated segmentation tool.

This study also included the publicly available dataset [16] that contained contrast-enhanced T1-weighted, high-resolution T2-weighted MR and VS contours of 242 patients from a single institution. Same as our data, these were the treatment planning images and target volumes for GK radiosurgery of VS. The MR images were acquired on a 1.5T Simens scanner. The T1W MR was obtained with a MPRAGE sequence with an in-plane resolution of 0.4 × 0.4 mm and a slice thickness of 1.0-1.5 mm. To develop a VS segmentation model for isotropic T1W MR, we halved the in-plane resolution and double the 1.5 mm cross-plane thickness to get a resolution of 0.8 × 0.8 × 0.75-1.0 mm. In all, the study employed data of T1W MR and VS contours of total 737 patients, 587 of which were used for model development (512 for training, 75 for validation), and 150 as an independent test set. Table 1 also show the summary of the data split in addition to the patient and tumor statistics.

### CNN architecture and training

**Fig. 1:**
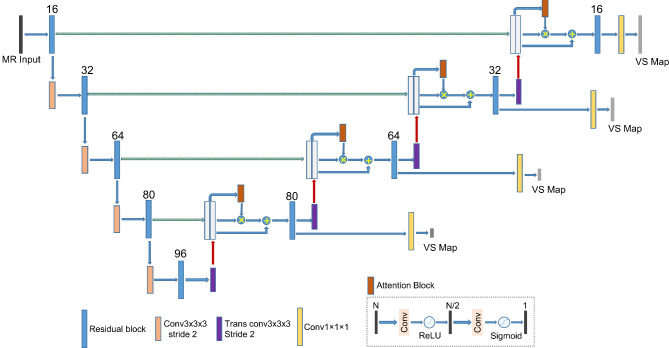
Architecture of the CNN model

The model followed the typical U-Net architecture [18] which learned 3D representative features along the encoding pathway and derived the segmentation map following the decoding pathway to the original resolution. Figure 1 shows the overall architecture of the CNN model with channel numbers of 16, 32, 64, 80 and 96 from the top to bottom layer. The network was built upon the ResNet block [19] which applied an additional skip connection on two convolution-normalization-ReLU units. The block used 3 × 3 × 3 kernel convolutions, batch normalization, and a dropout rate of 0.3.

Progressively halving the resolution through the encoding pathway was achieved by a 3 × 3 × 3 kernel convolution with a stride of 2. In the decoding pathway, a transpose convolution with a stride of 2 doubled the resolution while updating feature widths correspondingly. The up-sampled features were concatenated with the feature maps from the corresponding encoder level. A spatial attention module followed to grant higher important scores to the voxels within the tumor region while lowering the scores of the outside voxels [20]. The module consisted of two convolutions followed by a ReLU and Sigmoid activation function, respectively, to generate a spatial attention map. The map represented the possibility of each voxel belonging to the tumor target, and was directly supervised by a spatial attention loss in comparison with the truth segmentation. The attention map then scaled the concatenated features to focus more on the small target area than the large background.

The deep supervision mechanism [21] was utilized in the decoding pathway to regularize model learning at each layer of the U-Net. The deep supervision generated a segmentation map at each decoder level by using a 1 × 1 × 1 kernel convolution and a sigmoid activation function on the feature resulting from the ResNet block of the level. These output maps were connected to the loss functions that evaluated their distances from the ground truths that had been down-sampled to the corresponding resolutions. Thereby, deep supervision would ease the vanishing gradient problems in training of a deep model and drive the hidden layers to favorably learn discriminative features for segmentation [22]. The output at the last layer was the final segmentation map for the MR images.

Both the spatial attention loss and deep supervision loss were the conventional Dice loss [23] that assessed the dice overlap coefficient between the predicted maps and the ground truth segmentation. The Dice loss can effectively alleviate the imbalance of the target and background voxels as the VS only accounted for a very small portion of the whole images. The total segmentation loss for training minimization was unweighted sum of these losses at the multiple levels of the decoder. Additionally, L2 regularization for the model parameters was included in the loss function with a weighting of 1e-7 to reduce overfitting during the model training.

The model was implemented using Python with MONAI and PyTorch framework on a high-performance computing cluster with16gb NVidia Tesla V100 GPUs. Each MR volume was preprocessed independently by intensity normalization which was to subtract the mean and divide by standard deviation of the volume. Data augmentation including rand affine transformation, random image contrast adjustment and Gaussian noise adding were applied to improve the model robustness. Due to the memory limitation, the model was trained on image patches of a size of 128 × 128 × 96 with a batch size of 1. The training patches were extracted by random negative-positive crop of the images to improve class balance of the samples. The model training used the Adam optimization with a learning rate beginning with 0.003. The learning rate was halved for every 100 epochs in the first 200 epochs, and then for every 50 epochs for a total 300 epochs. The hyper-parameters were experimentally determined with the validation data. Lastly, the CNN achieving the best performance on the validation dataset was the final model for VS segmentation.

### Model testing and evaluation

The test images were preprocessed with intensity normalization, and then put to the CNN using the sliding window approach with a window size of 128 × 128 × 96 and 25% overlap of the windows. The binary tumor segmentation was obtained by applying a threshold of 0.5 to the resultant map. The model performance was evaluated on the test data using the Dice similarity coefficient (DSC), 95% Hausdorff distance (HD95), average symmetric surface distance (ASSD), and relative absolute volume difference (RAVD) between the predicted and ground-truth segmentations. DSC measures the spatial overlap between two segmentations, ranging from 0 for no overlap to 1 for perfect matching. HD95 quantifies the maximal distances of the border voxels of one segmentation to the other surface, but eliminates the impact of a small set of outliers. Instead, ASSD calculates the average of the border voxel distances, i.e., the mean distance of the two segmentation surfaces. RAVD measures the percentage absolute difference between the volumes of two segmentations, indicating the accuracy of using the automatic segmentation to measure tumor volume size.

We trained the model on the combined data from our institution and the public dataset. The metrics of performance assessment were evaluated on individual institution and total testing datasets. The performances of the model assessed between the two institutional datasets were compared by unpaired t-test of the resulting DSCs. Meanwhile, ablation experiments of training and testing the CNN without the spatial attention (SA) or the deep supervision (DSV) were performed on the same set of data.

## Results

**Fig. 2:**
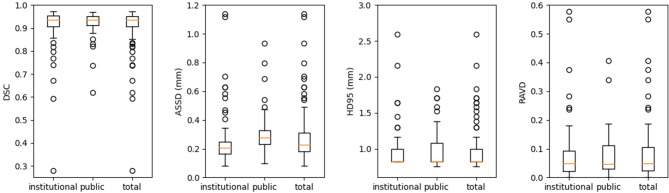
Box-and-whisker plots of the metrics to evaluate model performances on the testing dataset

**Fig. 3:**
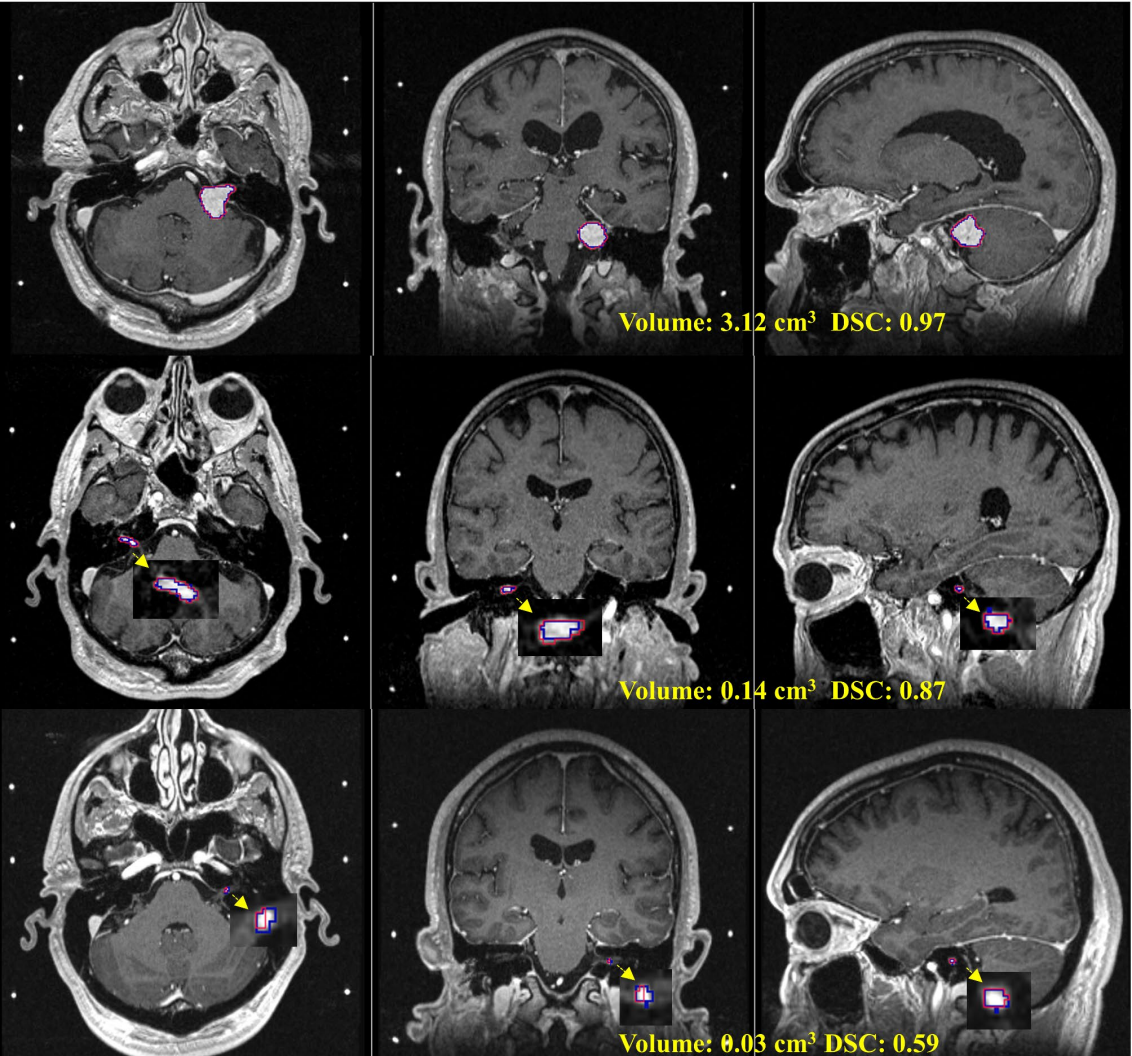
Three examples of the automatic segmentation results. Row: VS with different sizes, the last is the smallest in total dataset; Column: axial, coronal, sagittal slices of the MRI. Blue curve: ground truth tumor contours; Red curve: model segmentation results

Figure 2 demonstrates quantitative evaluations of the model applied to the testing dataset. On 150 patient data from both institutions, the mean (± SD) DSC was 0.91 ± 0.08, the mean surface distance was 0.3 ± 0.4 mm, and the HD95 indicated that 95% border voxels of the resulting contours were within 1.3 ± 1.6 mm from the ground truth surfaces. The model estimated tumor volumes with 9% ± 15% difference from the truth. Separately, the DSC was 0.91 ± 0.09 on our institutional MR and 0.92 ± 0.06 on the public dataset, showing similar performance between the two institution data (p = 0.66). Figure 3 provides illustrative examples of the CNN segmentation results with different performances, including for the smallest tumor with a size of 0.03cm^3^.

The CNN model detected VS in all the testing cases, but the models without the spatial attention or deep supervision missed the smallest tumor (Fig. 3, last row). Table 2 compares these models on the testing data excluding the smallest tumor, demonstrating incremental improvement of the segmentation accuracy by incorporating the two mechanisms. Paired t-tests on the DSCs of the testing cases shows the SA + DSV model has near significant difference from the DSV model (p = 0.05) in the model performances, but no significant difference observed with the SA model (p = 0.21).

**Table 2:** Comparisons between the proposed model and the models without spatial attention (SA) or deep supervision (DSV). The smallest tumor was excluded as the SA or DSV-only models failed to detect it

		Institutional Data	Public Data	Total Data
DSC	SA	0.901 ± 0.128	0.919 ± 0.060	0.907 ± 0.110
	DSV	0.905 ± 0.122	0.922 ± 0.070	0.910 ± 0.108
	SA + DSV	0.916 ± 0.081	0.918 ± 0.060	0.917 ± 0.074
ASSD (mm)	SA	0.51 ± 1.73	0.37 ± 0.34	0.46 ± 1.43
	DSV	0.35 ± 0.62	0.35 ± 0.45	0.35 ± 0.57
	SA + DSV	0.30 ± 0.42	0.36 ± 0.34	0.32 ± 0.40
HD95 (mm)	SA	2.13 ± 7.10	1.39 ± 1.83	1.88 ± 5.90
	DSV	1.38 ± 1.72	1.38 ± 2.42	1.38 ± 1.98
	SA + DSV	1.26 ± 1.42	1.36 ± 2.06	1.29 ± 1.66
RAVD	SA	9.4 ± 15.1	10.8 ± 19.5	9.9 ± 16.7
	DSV	9.5 ± 14.1	11.1 ± 25.5	10.0 ± 18.7
	SA + DSV	8.0 ± 11.1	11.2 ± 19.8	9.1 ± 14.7

**Fig. 4:**
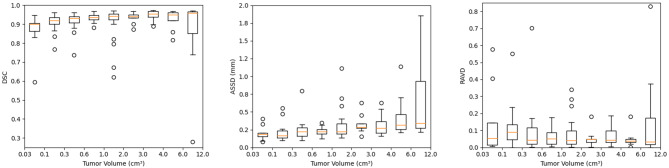
Model performances on segmentation of VS with different tumor sizes

**Fig. 5:**
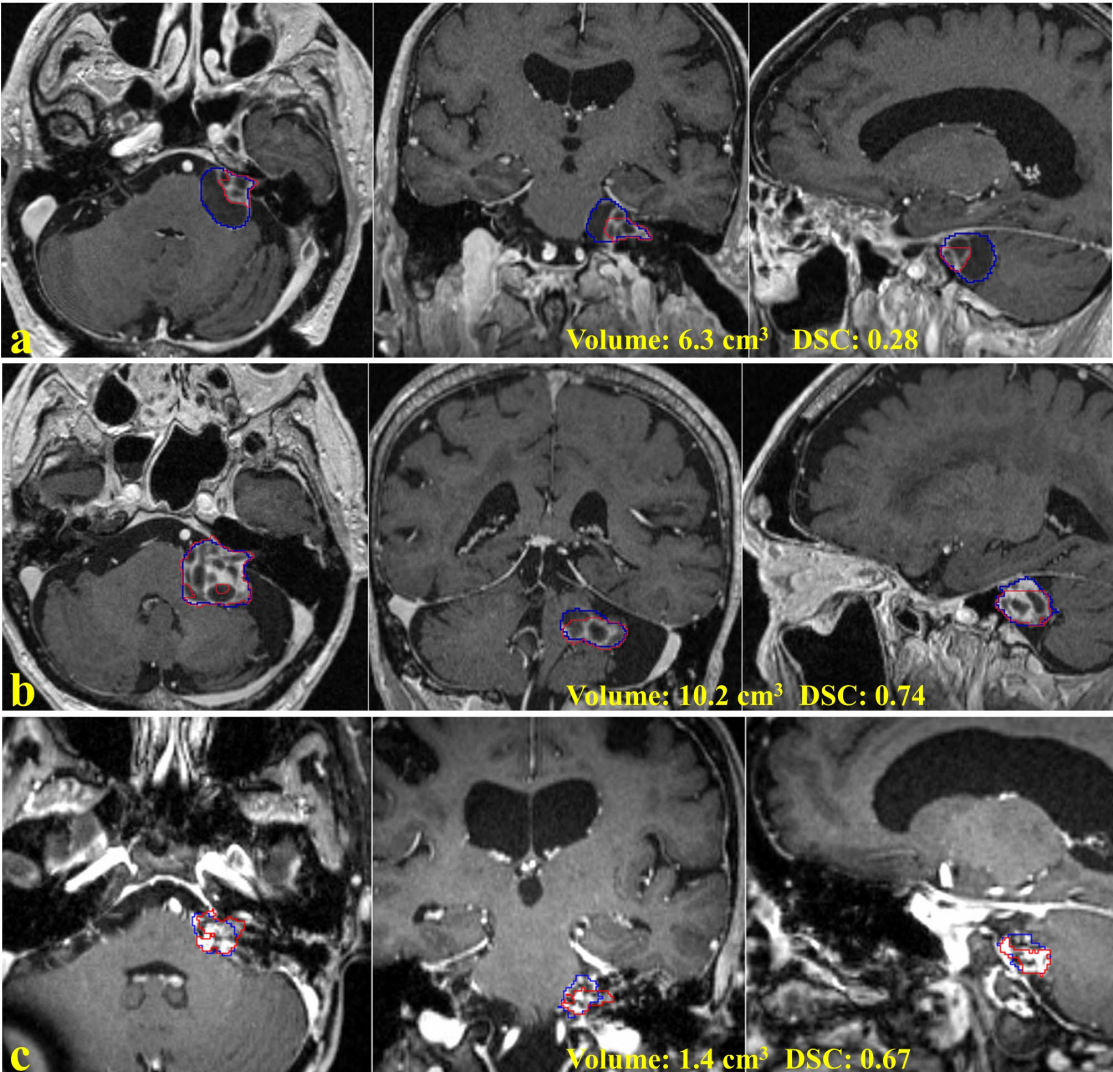
Three outliers of the automatic segmentation on the testing data. Row: (a, b) mixed cystic VSs; (c) post-surgical resection tumor. Column: axial, coronal and sagittal slices of MRI. Blue curve: ground truth tumor contours, Red curve: model segmentation results

The testing data was stratified into groups with different tumor volumes to understand the dependence of the model performance on tumor sizes. The mean DSC were 0.86 ± 0.10 for tumor size < 0.1 cm^3^ (n = 9), 0.92 ± 0.05 for size between 0.1 and 6 cm^3^ (n = 132). However, the mean DSC became 0.85 ± 0.21 for size > 6cm^3^ (n = 9), while the median DSC was 0.96 (25–75%: 0.85–0.97). Figure 4 indicates the average DSC was significantly distorted by the outliers which are showed in Fig. 5. The outliers in the size group were mixed cystic tumors (Fig. 5a, b) with substantial inhomogeneities of contrast enhancement on MRI. Figure 5c is a post-surgical resection tumor that yielded a low DSC. Clinically, the image interpretation of VS after resection is often challenging. The model achieved a mean DSC of 0.89 ± 0.07 on 15 testing patients of the institution who had prior surgery before GKRS.

## Discussion

Automated segmentation of VS on anisotropic MR have been addressed by a number of studies using DL techniques. Shapey et al. achieved a mean DSC of 0.93 on T1W MR with an in-plane resolution of 0.4 × 0.4 mm and a slice thickness of 1.5 mm [14]. Lee et al. obtained an average DSC of 0.90 using both T1W and T2W MR with a resolution of 0.5 × 0.5 × 3 mm for both sequences [15]. As the inter-slice thickness was much greater than the intra-slice resolution, both CNNs began with 2D convolution and down-sampling layers that generated isotropic features for following 3D feature extraction. Recently, Neve et al. built a 3D CNN model on T1W MR with a resolution of 0.35 × 0.35 × 1.0 mm, and obtained a DICE of 0.92 on 47 test cases of the institution [24]. However, applying the model on the public dataset yielded an average DSC of 0.88. It was suggested that the reason was the study contoured VS by radiologists while the public data (and ours) contoured GTV conservatively for GK treatment planning [24]. Our clinical workflow acquires high-resolution near-isotropic (0.8 × 0.8 × 1 mm) T1W MR for treatment planning. We instead employed 3D U-Net to exploit 3D features from the beginning. The CNN model achieved a mean DSC of 0.91 but on isotropic MR. The studies of Shapey et al.[14] and Neve et al.[24] had another physician perform the contouring on their anisotropic MR and reported DSCs of 0.94 and 0.91, respectively, for the second human annotations. Given a margin of 5% for DICE score, and the fact that the tumors of the published studies had 2–3 times more voxels than that if on our isotropic images, our model achieved a performance equivalent to those of the reported DL methods and human annotations, fitting the need of VS segmentation for isotropic imaging protocol.

The proposed model was a 3D U-Net built upon residual blocks. U-Net extracts and concatenates 3D features at different resolution scales, generates segmentation map by classifying each voxel based on a large number of global and local features [18]. The residual block adds skip connection to the convolutions, simply but very effectively eases the difficulties of exploding and vanishing gradients in deep neural network training [19]. Furthermore, the deep supervision compares the outputs at each level of the decoder. It further eases the vanishing gradient problem and enhances the learning of discriminative features at the hidden layers [21]. Additionally, the spatial attention module explicitly drives the learning to the target voxels, focusing on the small target region in the much larger surrounding area. The mechanism has been successfully used to address the challenge of small tumor size [14, 20]. Table 2 demonstrated both the spatial attention and deep supervision contribute to the performance of the model, particularly allowing detection of the smallest tumor (Fig. 3).

Our clinical GKRS workflow acquires contrast-enhanced high resolution isotropic T1W MR for target delineation and treatment planning, and low resolution (1.5 × 1.5 × 1.5-3.0 mm) T2W MR and other sequences to provide complementary information. Clinical practice of the team over the past few decades have proved the efficacy and efficiency of the imaging protocol. The accuracy of VS segmentation on high resolution (0.5 × 0.5 × 1.0-1.5 mm) T2W MR was lower than that achieved on contrast enhanced T1W MR [20], and the improvement using both T1W and T2W MR was marginal [14]. The recent study examined DL segmentation on T2W MR with an even higher resolution (0.3 × 0.3 × 0.6 mm), and reported a mean DSC of 0.87 [24]. Acquiring such high resolution MR would increase scan time. Current study aimed for automatic segmentation on contrast-enhanced T1W MR, facilitating target contouring for radiotherapy treatment planning.

Dramatic decreases in the performance of DL-based detection and segmentation of brain metastases occurred at lesion size less than 0.1 cm^3^ [25]. This study has detected every tested tumor including the smallest volume of 0.03 cm^3^. The median DSC was 0.90 when VS size was smaller than 0.1 cm^3^, exhibiting the accuracy of the proposed model for small VS. Better segmentation results were achieved when the tumor size increased. However, outliers manifesting the worst performances occurred in the results. These tumors were post resection or cystic tumor, whose MR intensities and appearance were different (Fig. 5). The cystic region appears high intensity in T2W MRI. Incorporating T2W MRI [15] could be helpful to address the deficiency of the current model. Our future work will improve the model by using multi-parametric MRI including T1W, T2W and others.

While the current study were developed on a sizeable dataset from two institutions, the DL model is still limited by the data size. The two dataset represent the standard clinical practices of delineating VS for GKRS. They used institution-specific imaging sequences and physician-dependent tumor annotations. Using two independent datasets potentially improves the generalization and robustness of the model. Nevertheless, these were two uniform datasets, consequently, the model need further training and evaluation on MR images acquired differently. Furthermore, using data only prior to treatment is another limitation to this study. Longitudinal change of the tumor volume over time is a decisive factor in management of VS patient post treatment. The size of a VS is currently quantified by the lesion’s maximal extrameatal linear dimension [6], which seems not as reliable or sensitive as the lesion 3D volume measurement [7]. With future study of the model on follow-up MRI, this tool could enable accurate and readily tumor volumetry during the long-term follow-up of patients after treatment.

## Conclusions

We developed a CNN model to automatically segment VS on the contrast enhanced T1-weighted MR with isotropic resolutions. The model achieved good performances in VS segmentation and volumetry on a large dataset from two institutions. The proposed method potentially facilitates VS radiosurgery workflow. Future study of the model on follow-up MR will establish a tool to improve long-term management of VS after treatment.

## Data Availability

The datasets used and/or analyzed during the current study are available from the corresponding author on reasonable request.

## Abbreviations

VS: Vestibular Schwannomas
SRS: Stereotactic radiosurgery
GK: Gamma Knife
GKRS: Gamma Knife Radiosurgery
GTV: Gross tumor volume
MRI: Magnetic Resonance Imaging
T1W MRI: T1-weighted MRI
DL: Deep learning
DNN: Deep neural network
CNN: Convolutional neural network
DSC: DICE similarity coefficient
HD95: 95% hausdorff distance
ASSD: Average symmetric surface
RAVD: Relative absolute volume difference
